# Kirkiin: A New Toxic Type 2 Ribosome-Inactivating Protein from the Caudex of *Adenia kirkii*

**DOI:** 10.3390/toxins13020081

**Published:** 2021-01-22

**Authors:** Massimo Bortolotti, Stefania Maiello, José M. Ferreras, Rosario Iglesias, Letizia Polito, Andrea Bolognesi

**Affiliations:** 1 Department of Experimental, Diagnostic and Specialty Medicine-DIMES, General Pathology Section, Alma Mater Studiorum—University of Bologna, 40126 Bologna, Italy; massimo.bortolotti2@unibo.it (M.B.); stefaniamaiello@libero.it (S.M.); andrea.bolognesi@unibo.it (A.B.); 2 Department of Biochemistry and Molecular Biology and Physiology, Faculty of Sciences, University of Valladolid, E-47011 Valladolid, Spain; josemiguel.ferreras@uva.es (J.M.F.); riglesia@bio.uva.es (R.I.)

**Keywords:** *Adenia*, apoptosis, kirkiin, lectins, neuroblastoma, ribosome-inactivating proteins, ricin, toxic enzymes

## Abstract

Ribosome-inactivating proteins (RIPs) are plant toxins that irreversibly damage ribosomes and other substrates, thus causing cell death. RIPs are classified in type 1 RIPs, single-chain enzymatic proteins, and type 2 RIPs, consisting of active A chains, similar to type 1 RIPs, linked to lectin B chains, which enable the rapid internalization of the toxin into the cell. For this reason, many type 2 RIPs are very cytotoxic, ricin, volkensin and stenodactylin being the most toxic ones. From the caudex of *Adenia kirkii* (Mast.) Engl., a new type 2 RIP, named kirkiin, was purified by affinity chromatography on acid-treated Sepharose CL-6B and gel filtration. The lectin, with molecular weight of about 58 kDa, agglutinated erythrocytes and inhibited protein synthesis in a cell-free system at very low concentrations. Moreover, kirkiin was able to depurinate mammalian and yeast ribosomes, but it showed little or no activity on other nucleotide substrates. In neuroblastoma cells, kirkiin inhibited protein synthesis and induced apoptosis at doses in the pM range. The biological characteristics of kirkiin make this protein a potential candidate for several experimental pharmacological applications both alone for local treatments and as component of immunoconjugates for systemic targeting in neurodegenerative studies and cancer therapy.

## 1. Introduction

Ribosome-inactivating proteins (RIPs) are toxic enzymes widely distributed in the plant kingdom, but also present in some fungal and bacterial species [1,2,3]. RIP-containing plants are largely used in folk and traditional medicine worldwide, and several derivatives from these plants are still employed for the treatment of numerous pathologies [4,5]. RIPs are classified as rRNA N-glycosylase (EC 3.2.2.22), as they recognize a specific and universally conserved region of 14 nucleotides on 28S rRNA, splitting the N–C glycosidic bond between a specific adenine and its ribose in the sequence GAGA on the rRNA. In the case of rat liver ribosomes, this site is A4324 and is positioned within a single-stranded loop called sarcin-ricin (SRL) [6]. After adenine removal, the apurinic site does not allow the GTPase-dependent binding of elongation factor-1 (EF-1) and elongation factor-2 (EF-2) to the 60S subunit of the ribosome, thus blocking the translation [7].

Further studies showed that RIPs can deadenylate a range of polynucleotides, such as DNA, tRNA, mRNA, and viral RNA and the term polynucleotide:adenosine glycosidases was proposed [8,9]; afterwards, the activity was better defined as adenine polynucleotide glycosylase [10].

RIPs are structurally divided into two main groups: type 1 RIPs, characterized by a single polypeptide chain of about 30 kDa with enzymatic activity, and type 2 RIPs with molecular weight of about 60 kDa, consisting of an enzymatically active A chain, similar to type 1 RIPs, linked through a disulfide bond to a B chain with lectin properties. The B chain has strong affinity for sugar moieties on cell surface and can facilitate the entry of the toxin into the cell, thus conferring to many type 2 RIPs high cytotoxicity [11,12,13].

Several studies indicate that RIPs have a role in protecting plants from viral and fungal infection or in plant senescence [14]. However, their action in plants defense from pathogens is not still clear. In general, type 2 RIPs seem to be more active on animal ribosomes, whereas type 1 RIPs have a wider specificity. This suggested that adenine polynucleotide glycosylase activity might be responsible for the antiviral action of RIPs [8].

To date, about 80 type 2 RIPs have been purified from a few plant genera [1,2]. In particular, RIPs purified from *Adenia* genus are among the most lethal plant toxins. In addition to modeccin and volkensin, extracted from the roots of *Adenia* (Modecca) *digitata* (Harv.) Engl and *Adenia volkensii* Harms, respectively [15,16], that are known for many years, two other potent toxins from the caudices of *Adenia lanceolata* Engl. (lanceolin) and *Adenia stenodactyla* Harms (stenodactylin) were subsequently described [17]. The high cytotoxicity of *Adenia* RIPs is probably due to their high-affinity cell binding, efficient endocytosis and intracellular routing, resistance to proteolysis, and, regarding stenodactylin, high accumulation into the cell [18]. *Adenia* toxins are retrogradely transported along peripheral nerves and in the central nervous system [19,20]; this property could have different medical and biotechnological applications in neurophysiology and for the experimental treatment of pain [21]. Moreover, because of their high cytotoxicity, RIPs can be used for pharmacological purpose, both native, for local–regional treatments, and as components of immunotoxins, for systemic therapy of cancer and other pathologies [22,23,24].

It has been reported that in a neuroblastoma cell line, stenodactylin induced multiple cell death pathways, involving apoptosis, necroptosis, and oxidative stress [25]. Moreover, stenodactylin elicited a quick stress response in leukemia cells, producing pro-inflammatory factors and oxidative stress, triggering apoptosis and other cell death pathways [26].

These peculiar characteristics of *Adenia* toxins prompted us to evaluate whether other species belonging to *Adenia* genus, i.e., *Adenia kirkii* (Mast.) Engl. (hereafter referred as *A. kirkii*), contain lectins or toxic RIPs structurally similar to others, already purified from the same genus, and possibly endowed of peculiar biological properties. In this study, a new toxic type 2 RIP, named kirkiin, was purified from the caudex of *A. kirkii*, and its biochemical, enzymatic, and cytotoxic properties were evaluated.

## 2. Results

### 2.1. Purification and Characterization of Adenia Kirkii Lectins

The extracts from *A. kirkii* caudex were purified by chromatography on an acid treated-Sepharose CL-6B column. The acidic treatment causes the exposure of galactose residues present in the cross-linked agarose matrix. In this manner, the stationary phase become able to bind lectins. This affinity chromatography method allows the one-step purification of lectins present in the crude extract. The lectin was eluted from the stationary phase with 0.2 M galactose. The purified lectin was assayed for the inhibition of protein synthesis in a rabbit reticulocyte lysate system (Table 1). *A. kirkii* lectin showed high enzymatic activity, with concentration inhibiting 50% of protein synthesis (IC_50_) values of 9.2 and 4.7 μg/mL for the non-reduced and reduced protein, respectively, comparable with those obtained with other RIPs purified from *Adenia* genus. Moreover, *A. kirkii* lectin had high agglutinating activity for human erythrocytes, showing a minimum concentration causing agglutination of 4.0 μg/mL, a value lower than that obtained with other *Adenia* RIPs.

**Figure 1:**
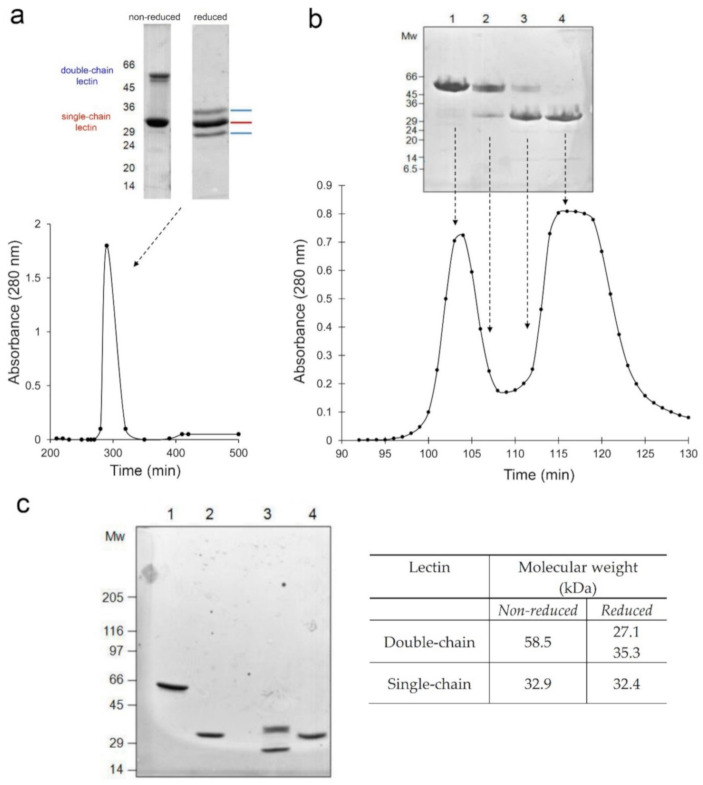
(**a**) Chromatography on acid-treated Sepharose CL-6B of *A. kirkii* extracts. Proteins were eluted with 0.2 M galactose in PBS. SDS-PAGE analysis of peak fractions under non-reducing and reducing conditions on 8–25% gradient polyacrylamide gel. (**b**) Chromatography by gel filtration on Sephacryl S-100 of acid-treated Sepharose CL-6B eluate. Proteins were eluted in PBS and peak fractions were analyzed on 8–25% gradient polyacrylamide gel. (**c**) SDS-PAGE of lectins under reducing and non-reducing conditions. Lane 1 and 2 correspond to the non-reduced high and low molecular weight lectins, respectively. Lanes 3 and 4 correspond to the reduced high and low molecular weight lectins, respectively. The electrophoresis was carried out on a 4–15% gradient polyacrylamide gel (staining with Coomassie Blue). Molecular weights of the standard are expressed in kDa. In table, molecular weights of each band, expressed in kDa, are reported after calculation by densitometric analysis of the gel.

As the lectin from *A. kirkii* showed strong toxicity, this prompted us to deepen the study of the new toxin. A further purification procedure was undertaken by chromatography on acid-treated Sepharose CL-6B. A single peak of protein material was eluted with 0.2 M galactose (Figure 1a), resulting in 107.9 mg of total proteins with RIP activity obtained from 100 g of fresh tissue. The yield of purification was 13.1% (Table 2). On gel electrophoresis (Figure 1a), the non-reduced proteins from *A. kirkii* gave two bands with relative mobility (Mr) of about 60 and 30 kDa, approximately. After reduction, proteins from *A. kirkii* showed three bands with Mr of about 30 kDa. This suggests that two lectins with different molecular weights are likely present in *A. kirkii* caudex.

Subsequently, a chromatography by gel filtration on Sephacryl S-100 was performed in order to separate the two lectins. The chromatography allowed the complete separation of the two lectins. As shown in Figure 1b, the acid-treated Sepharose CL-6B eluted peak was resolved into two well separated peaks, the first one corresponding to the high molecular weight lectin (double-chain lectin) and the second one to the low molecular weight lectin (single-chain lectin). The yield was approximately 14 mg of double-chain lectin and 36 mg of single-chain lectin per gram of tissue (see Table 2). Both lectins agglutinated human erythrocytes; the minimum agglutinating concentration being 175 and 2.9 μg/mL for double-chain and single-chain lectin, respectively. 

The fractions corresponding to each peak were collected and analyzed by SDS-PAGE on a 4–15% gradient gel in order to verify their purity (Figure 1c). High molecular weight lectin revealed the presence of a single band with Mr of 58.5 kDa under non-reducing conditions (Figure 1c, lane 1). After reduction with 2-mercaptoethanol, two bands of 27.1 kDa and 35.3 kDa were obtained (Figure 1c, lane 3). The low molecular weight lectin showed only one band of about 32 kDa both in non-reducing and reducing conditions (Figure 1c, lane 2 and lane 4, respectively).

### 2.2. Enzymatic Properties of Kirkiin

#### 2.2.1. Effect on Protein Synthesis

The effect of the purified lectins on mammalian ribosomes was evaluated in vitro in a cell-free system consisting of rabbit reticulocyte lysate, by assaying their inhibitory activity on protein synthesis. The two lectins were assayed both in native form and under reducing conditions, thus eliminating the possible steric hindrance given by B chain. As shown in Table 2, double-chain lectin strongly inhibited protein synthesis, with IC_50_ values of 7.4 μg/mL in the native status and of 1 μg/mL after reduction. Instead, single-chain lectin revealed a low inhibition activity with IC_50_ value greater than the highest tested dose (50 μg/mL). For this reason, we chose to continue the research only with the type 2 toxin, hereafter referred as kirkiin.

#### 2.2.2. rRNA N-Glycosylase Activity on Mammalian and Yeast Ribosomes

**Figure 2:**
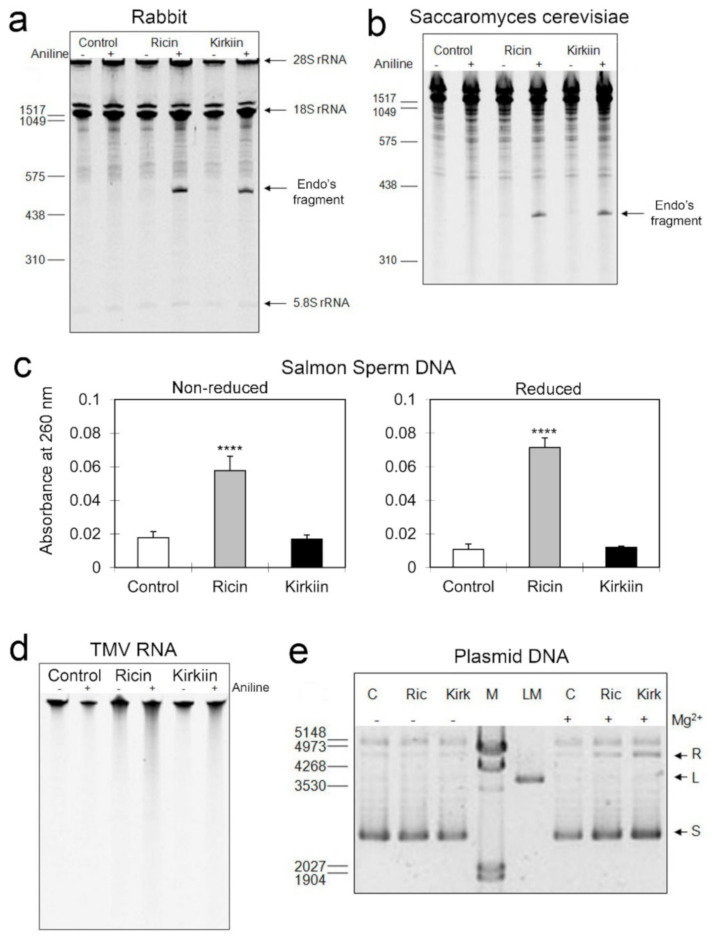
rRNA N-glycosylase activity of kirkiin and ricin on rabbit reticulocyte ribosomes (**a**) and on yeast ribosomes (**b**). Each lane contains 3 μg of RNA. The arrows indicate the 28S, 18S, and the 5.8S rRNAs, and the RNA fragments released as a result of RIP action after aniline acetate treatment at pH 4.5 (+). Numbers indicate the size of the standards in nucleotides. Adenine polynucleotide glycosylase activity of kirkiin and ricin on salmon sperm DNA (**c**) and on Tobacco Mosaic Virus RNA (**d**). In the first case, the amount of released adenine was determined by measuring the absorbance at 260 nm of the supernatant obtained by centrifugation of the samples. The results are the means of two independent experiments, each performed in duplicate. **** *p* < 0.0001, *t*-student test. In the second case, each lane contains 1 μg of RNA. The depurination activity was assayed after aniline acetate treatment at pH 4.5 (+). (**e**) Endonuclease activity of kirkiin (Kirk) and ricin (Ric) on supercoiled plasmid DNA (pCR 2.1) compared to control (C). Each lane contains 100 ng of plasmid DNA. The arrows indicate the supercoiled (S), the linear (L), and the relaxed (R) forms of the plasmid. Numbers indicate the size of the standards (M) in base pairs, and (LM) represents the linear form of the plasmid used as standard.

Kirkiin rRNA N-glycosylase activity was performed through RNA depurination assay of mammalian ribosomes, using rabbit reticulocyte lysate as substrate. The activity was compared to that of the most known type 2 RIP ricin from *Ricinus communis* L. seeds. Both kirkiin and ricin displayed the ability to depurinate mammalian rRNA evidenced by the release of the RNA fragment upon treatment with acid aniline (Endo’s fragment), which is diagnostic for RIP action on ribosomes [27]. No RNA fragment was observed in control samples and in samples treated in absence of aniline. These results confirm that inhibition of protein synthesis induced by kirkiin is related to its N-glycosylase activity on mammalian ribosomes (Figure 2a).

The effect of kirkiin was also assayed on ribosomes from *Saccharomyces cerevisiae*, which might be homologous to ribosomes from putative plant pathogens. As shown in Figure 2b, kirkiin and ricin displayed rRNA N-glycosylase activity on yeast ribosomes, as indicated by the release of the diagnostic fragment of 360 ± 30 nucleotides upon treatment with aniline acetate, in accordance with that expected for the SRL deglycosylation (368 nucleotides for yeast) [28]. Therefore, kirkiin is able to exert its action also on ribosomes of unicellular eukaryotes.

#### 2.2.3. DNA and RNA Adenine Polynucleotide Glycosylase Activity

Kirkiin adenine polynucleotide glycosylase activity was investigated on salmon sperm DNA (ssDNA) (Figure 2c) and on tobacco mosaic virus RNA (TMVR) (Figure 2d). Kirkiin activity was compared with that of ricin, which possesses a moderate activity. As shown in Figure 2c, kirkiin showed no significant activity on ssDNA both in reduced and non-reduced conditions. On TMVR also, kirkiin exhibited a marginal depurination upon treatment with acid aniline compared to control, resulting slightly less than that shown by ricin (Figure 2d).

#### 2.2.4. Endonuclease Activity on Supercoiled Plasmid DNA

The endonuclease activity of kirkiin was tested on pCR 2.1 plasmid, and it was compared with that of ricin. Both kirkiin and ricin promoted a slight conversion of supercoiled DNA into a relaxed form. This effect was dependent on magnesium ions, and the highest activity was observed at 5 mM of this ion (Figure 2e). Therefore, kirkiin as ricin showed a weak endonuclease activity and acted by cutting only one of the two helices of the plasmid DNA.

### 2.3. Immunological Properties

**Figure 3:**
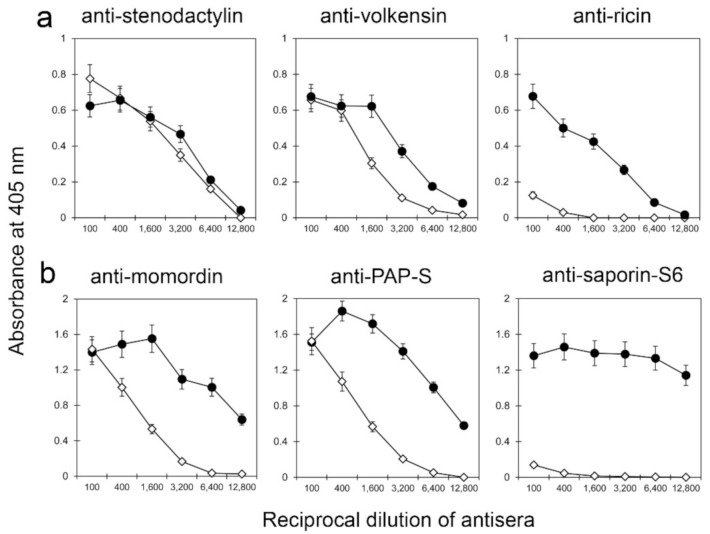
Enzyme-linked immunosorbent assay (ELISA) with (**a**) anti-type 2 and (**b**) anti-type 1 RIP sera. The values of absorbance at 405 nm are expressed in function of the reciprocal of serum dilution. Curves of the RIPs with the respective anti-sera are depicted with black symbols (●), while those related to kirkiin are represented in white symbols (◊). The results are the means of at least three independent experiments.

The immunological properties of kirkiin were tested with sera against different type 2 RIPs, i.e., the *Adenia* RIPs stenodactylin and volkensin, and ricin. Kirkiin highly cross-reacted with sera against the other two *Adenia* toxins, but no cross-reaction was evidenced with the serum against ricin (Figure 3a). Kirkiin was also tested with sera against some type 1 RIPs, i.e., momordin, pokeweed antiviral protein from seeds (PAP-S), and saporin-S6. Kirkiin showed partial cross-reactivity with anti-momordin and anti-PAP-S sera, while it did not react with the serum against saporin-S6 (Figure 3b).

### 2.4. Cytotoxic Effects

#### 2.4.1. Effect of Kirkiin on NB100 Protein Synthesis and Cell Viability 

**Figure 4:**
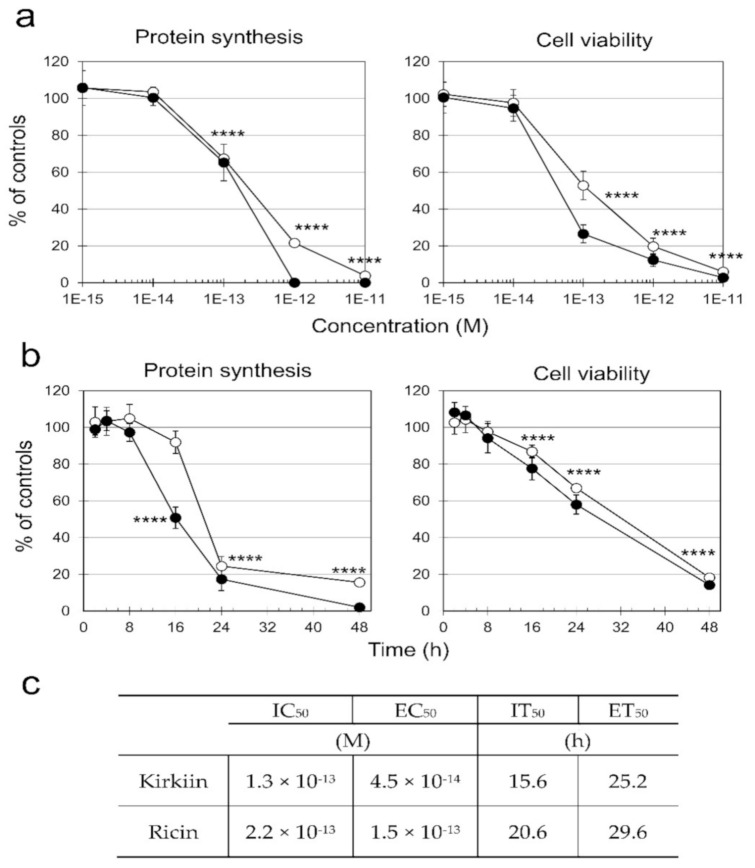
(**a**) Concentration–response curves. Comparison of protein synthesis and cell viability in NB100 cells treated with kirkiin (black symbols) or ricin (white symbols) for 72 h. Both parameters are expressed as percentage of controls. (**b**) Time–response curves. Protein synthesis and viability of NB100 cells treated with kirkiin or ricin (1 × 10^−11^ M) after the indicated times. (**c**) Table reports values of concentration and time that inhibit protein synthesis of 50% (IC_50_ and IT_50_, respectively), and values of concentration and time that reducing cell viability of 50% (IT_50_ and ET_50_, respectively). Protein synthesis inhibition was evaluated measuring the ^3^H-leucine incorporation in the neosynthesized proteins. Viability was evaluated using a colorimetric assay based on MTS reduction. The results are the means of three independent experiments, each performed in triplicate, and are represented as percentage of control values obtained from cultures grown in the absence of RIP. **** *p* ≤ 0.0001, ANOVA/Bonferroni, followed by comparison with Dunnett’s test.

Cytotoxic effects of kirkiin were compared to ricin and evaluated as protein synthesis inhibition and cell viability reduction in NB100 cells derived from a human neuroblastoma. Cells were treated with scalar concentrations of RIPs, ranging from 1 × 10^−15^ to 1 × 10^−11^ M for 72 h, and protein synthesis was assessed by incorporation of ^3^H-leucine into the new synthesized proteins. Kirkiin and ricin were extremely cytotoxic, showing IC_50_ values of 1.3 × 10^−13^ and 2.2 × 10^−13^ M, respectively. Nevertheless, kirkiin showed greater efficacy than ricin in inhibiting protein synthesis completely. At 1 × 10^−12^ M concentration, kirkiin was able to completely inhibit protein synthesis, whereas the same effect was reached by ricin at 1 × 10^−11^ M (Figure 4a,c).

In cell viability experiments at 72 h, both ricin and kirkiin completely killed all tested cells at 1 × 10^−11^ M. The concentration that reduces the cell viability of 50% (EC_50_, effective concentration fifty) was 4.5 × 10^−14^ and 1.5 × 10^−13^ M for kirkiin and ricin, respectively (Figure 4a,c). Because both toxins at 1 × 10^−11^ M resulted in the ability to completely inhibit protein synthesis and reduce cell viability after 72 h incubation, this concentration was chosen for further cytotoxicity experiments carried out in a time range from 2 to 48 h. Time-response curves (Figure 4b) showed that protein synthesis inhibition was faster than cell viability reduction; the time required to inhibit protein synthesis of 50% (IT_50_) (15.6 and 20.6 h for kirkiin and ricin, respectively) was shorter than that required to reduce cell viability of 50% (ET_50_, effective time fifty) (25.2 and 29.6 h for kirkiin and ricin, respectively, as shown in Figure 4c). 

#### 2.4.2. Evaluation of Apoptosis Induced by Kirkiin in NB100 Cells

**Figure 5:**
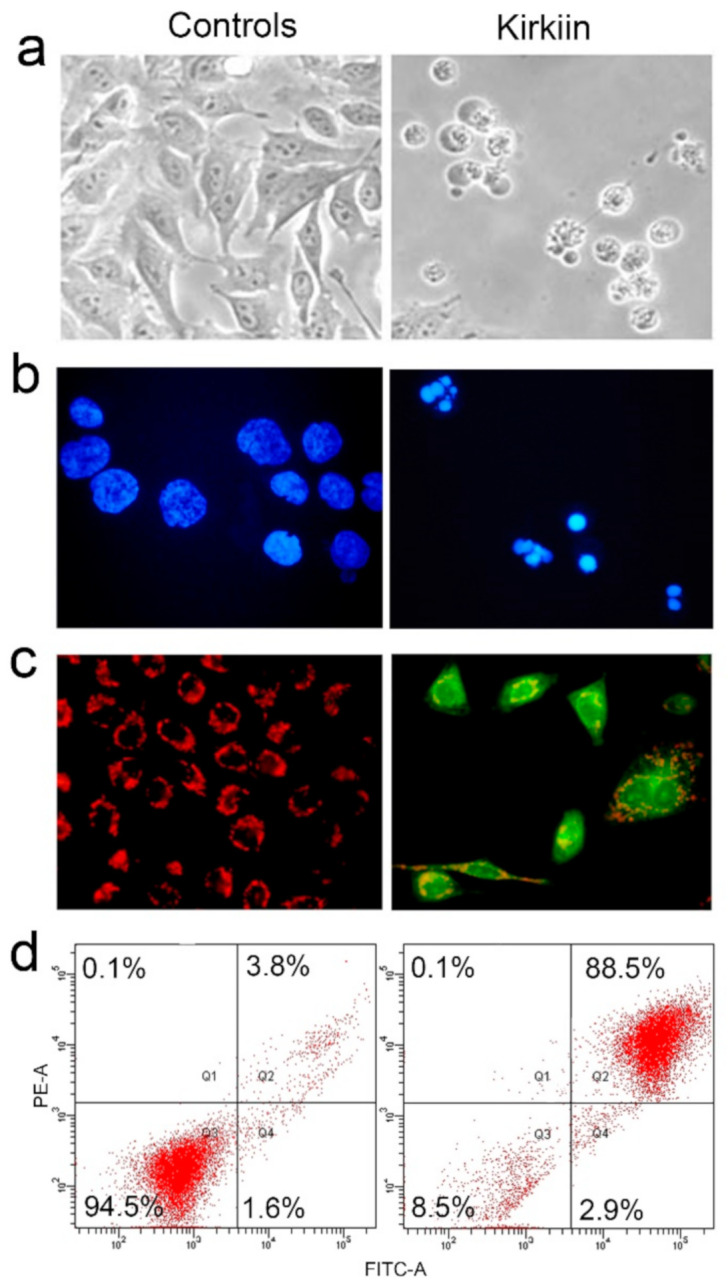
Induction of apoptosis. NB100 untreated (controls) or treated cells with kirkiin 1 × 10^−11^ M for 48 h were checked for (**a**) cell morphology through phase-contrast microscopy (600× magnification); (**b**) nuclear morphology through fluorescence microscopy after DAPI staining (600× magnification); (**c**) mitochondrial transmembrane potential dissipation through JC-1 staining and an analysis in fluorescence microscopy (600× magnification); (**d**) induction of necrosis/apoptosis by Annexin V-EGFP/PI double staining, followed by flow cytometry analysis. Representative plots of Annexin V (FITC channel)/PI (PE channel).

In order to evaluate the involvement of apoptosis, we examined the presence of cellular and nuclear morphological changes in NB100 cells treated for 48 h with kirkiin at 1 × 10^−11^ M concentration, using phase-contrast and fluorescence microscopy, respectively. Morphological characteristics of apoptosis were present in treated cells, such as cell shrinkage, loss of contact with adjacent cells, formation of cytoplasmic protrusions, and apoptotic bodies (Figure 5a). The staining of NB100 cells with DAPI showed that kirkiin intoxication induced a reduction of cell density and an increase of pyknotic and fragmented nuclei (Figure 5b). A typical feature of programmed cell death is the disruption of active mitochondria, which consists in changes in the membrane potential (Δψm) and alterations of the mitochondria redox state. Alterations of Δψm were detected through fluorescence in cells exposed to kirkiin, after staining with JC-1. Untreated cells showed a strong red fluorescence due to the characteristic J-aggregates in the mitochondria, indicating intact Δψm. In cells treated with kirkiin, JC-1 remained in the monomeric form, yielding green fluorescence and indicating dissipation of the Δψm. These results confirmed that cells undergo programmed cell death after kirkiin intoxication and that mitochondria are involved (Figure 5c).

Apoptosis was also evaluated by double staining with Annexin V-EGFP/Propidium iodide (PI) through flow cytofluorimetric analysis of NB100 cells, in order to quantify the percentage of apoptotic cells and evaluate the eventual involvement of necrosis. PI-positive cells (necrotic cells) are in the upper left quadrant, while apoptotic cells are in the upper (late apoptosis) and lower (early apoptosis) right quadrants. After 48 h of intoxication with the RIP, 88.5% of treated cells were in late-stage apoptosis (Figure 5d). No significative involvement of necrosis was detected after kirkiin intoxication.

**Figure 6:**
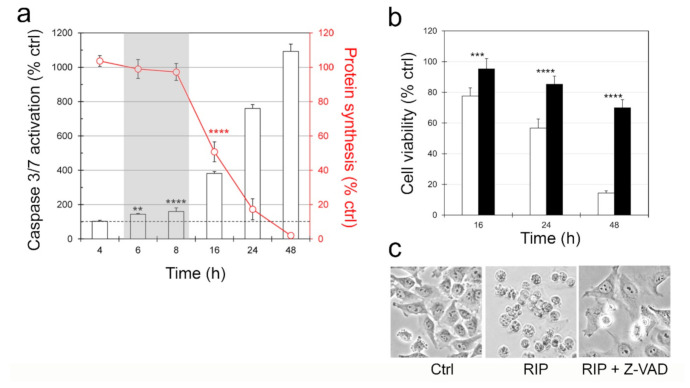
Involvement of caspase-dependent apoptosis. (**a**) Caspase 3/7 activation. Caspase activation (columns) was compared with protein synthesis (red line). Shaded area highlights the time range in which protein synthesis is not inhibited and caspases are significantly activated. Results were expressed as percentage of control values obtained from cultures grown in the absence of RIP. The results are the means of three independent experiments, each performed in triplicate (** *p* < 0.01, **** *p* < 0.0001, ANOVA/Bonferroni test). (**b**) Protection obtained by Z-VAD. NB100 cells were treated with 1 × 10^−11^ M kirkiin, alone (white columns) or preceded by a 3-h preincubation with 100 µM Z-VAD (black columns). The viability was measured after the indicated times. The statistical analysis was performed using ANOVA/Bonferroni test (confidence range 95%; *** *p* ≤ 0.001; **** *p* ≤ 0.0001. (**c**) Cell morphology was evaluated at 48h intoxication (400× magnification).

To determine the involvement of caspase-dependent apoptosis and to understand the correlation between protein synthesis and apoptosis, caspase 3/7 activation and protein synthesis inhibition were measured in NB100 cells exposed to kirkiin 1 × 10^−11^ M, in a time range from 4 to 48 h. As shown in Figure 6a, a strong time-dependent activation of caspase 3/7 was observed, which became significant compared to control after 6 h of treatment (143%) and highly significant after 8 h (160%). The level of caspase activity grew exponentially over time, reaching 1093% after 48 h.

The activation of caspases does not proceed in parallel with the inhibition of protein synthesis. Actually, the inhibition of protein synthesis became significant starting from 16 h of treatment (50% of controls) (Figure 6a, shaded area). These data indicate that protein synthesis and apoptosis are independent events.

To confirm the role of caspase-dependent programmed cell death, the pan-caspase inhibitor Z-VAD was used to selectively inhibit the apoptotic pathway. NB100 cells were pretreated and maintained in 100 µM Z-VAD, and the cell viability was determined after different incubation times with kirkiin (16, 24 and 48 h). As shown in Figure 6b, Z-VAD was able to significantly rescue NB100 cells from death at all the tested times, suggesting the involvement of caspase-dependent cell death. After 48 h-kirkiin intoxication, cells pre-treated with Z-VAD showed a viability of 69.9% versus 14.4% of viability in cells not pre-treated with Z-VAD. These results were confirmed by morphological analysis, showing that after 48 h of intoxication, the most of cells pre-treated with Z-VAD had morphological characteristics similar to those of untreated cells (Figure 6c).

## 3. Discussion

*A. kirkii* is a plant spread in Kenya, eastern Tanzania, and Zanzibar with typical glandular-shaped leaves, green flowers, and a caudex as reserve organ, situated at the base of the plant [29].

In the present study, we demonstrate that *A. kirkii* caudex contains a high amount of two lectins that have the characteristics of galactose-specific lectins. The lower molecular weight lectin, in SDS-PAGE gel, showed only one band of 32 kDa both in reducing and non-reducing conditions, and it did not inhibit protein synthesis in a cell free system; this is compatible with a single-chain lectin. The presence of non-toxic lectins in *Adenia* plants has been already described [17]. The higher molecular weight lectin showed one band of about 60 kDa in non-reducing conditions and two bands of 27 and 35 kDa in reducing conditions. Based on the data reported in literature, the A chain of type 2 RIPs weights about 20–30 kDa, whereas the B chain about 30–35 kDa [30]. Therefore, we can assume that the two bands of approximately 27 and 35 kDa represent the A chain and the B chain of a type 2 RIP, respectively.

Both lectins agglutinated erythrocytes; in particular, single-chain lectin showed higher hemagglutination activity than double-chain lectin, probably due to the absence of the steric hindrance of A chain.

Double-chain lectin, named kirkiin, showed a strong inhibition of protein synthesis, displaying IC_50_ values of 7.4 μg/mL in the native status and of 1 μg/mL under reducing conditions. These results were comparable to those obtained for other *Adenia* RIPs already studied that showed IC_50_ values in the range of 2.4–7.5 μg/mL under non-reducing conditions and of 0.4–1.2 μg/mL under reducing conditions [31].

RIPs are commonly known as plant toxins able to recognize and remove a specific adenine from the universally conserved SRL of the 28S rRNA [6]. Kirkiin displayed rRNA N-glycosylase activity against mammalian ribosomes, as indicated by the RIP diagnostic RNA fragment upon treatment with acid aniline [27]. This result confirms that kirkiin ability to inhibit protein synthesis is related to the N-glycosylase activity on mammalian ribosomes. As several evidences suggest that rRNA N-glycosylase activity might play a role in plant defense [32], for example against fungi, the effect of kirkiin was assayed on ribosomes from *Saccharomyces cerevisiae*, which might be homologous to ribosomes from putative plant pathogens. Kirkiin displayed rRNA N-glycosylase activity on yeast ribosomes, as indicated by the release of the diagnostic fragment [33] upon treatment with aniline acetate. Therefore, as kirkiin showed ribosome-inactivating activity on unicellular eukaryotes, it might enter into the fungal cells and inactivate their ribosomes, avoiding the propagation of the pathogen.

Many RIPs are potent inhibitors of animal and/or plant viruses, although the mode of action for the antiviral activity is still not clear [34]. The discovery of a depurinating activity of RIPs on viral RNA allowed hypothesizing a possible use of RIPs as antiviral agents [35]. RIPs have shown a very variable activity on different types of nucleic acids. Adenine polynucleotide glycosylase activity of all toxic type 2 RIPs is significantly lower than type 1 RIPs [8]. No significant activity was detected with kirkiin on viral RNA and eukaryotic DNA with respect to ricin, although the latter has an adenine polynucleotide glycosylase activity substantially lower than type 1 RIPs. Kirkiin did not increase its activity after the reduction of the interchain disulphide bridge, according to what already observed with toxic type 2 RIPs (except for ricin) [36]. Endonuclease activity on plasmid DNA was reported for some RIPs, promoting the conversion of the plasmid from the supercoiled form to the relaxed or linear one [37]. Kirkiin showed a weak activity against supercoiled plasmid DNA. This ability can be important in order to understand the possible biological roles of RIPs, for example in plant defense against pathogenic micro-organisms or viruses. These data indicate that kirkiin, as many other type 2 RIPs, shows high toxicity to mammalian and yeast ribosomes, but slight or no adenine polynucleotide glycosylase activity on other nucleotide substrates.

As RIPs are highly immunogenic, the cross-reactivity between kirkiin and some antibodies-containing sera against various double-chain and single-chain RIPs was evaluated. This analysis may be of interest in order to identify toxins useful for prolonged therapeutic treatment with immunotoxins. In fact, it is possible to reduce the immune response in cancer therapy by varying the type of toxin and prolong the use of RIPs, preserving their therapeutic efficacy. Kirkiin highly cross-reacted with sera against *Adenia* toxins. This strong interaction is not surprising, since all these toxins have been purified from plants belonging to the *Adenia* genus and have a high homology in their amino acid sequences. Kirkiin partially cross-reacted with sera against type 1 RIPs momordin and PAP-S, while it did not react with anti-ricin and anti-saporin-S6 sera. This result represents a remarkable advantage, as ricin and saporin-S6 are the RIPs most used as components of immunoconjugates [38,39,40]. This is of great interest in prospecting the use of an immunotoxin containing kirkiin A chain in prolonged therapeutic treatments in substitution to immunotoxins containing ricin A chain or saporin-S6.

In order to clarify pathogenetic mechanisms of kirkiin intoxication, inhibition of protein synthesis and cell toxicity were tested on NB100 cells. This cell line was chosen because in previous experiments it resulted very sensitive to *Adenia* RIPs [17,25]. Moreover, NB100 cells could represent a good in vitro model for future neurophysiological studies with kirkiin. Kirkiin resulted very efficient in cell protein synthesis inhibition and cell killing experiments, showing IC_50_ and EC_50_ values comparable to those observed with ricin and other *Adenia* RIPs [17,25]. In time-course experiments, the effects of kirkiin on protein synthesis and viability were examined, showing that the inhibition of protein synthesis precedes the loss of cell viability. Actually, at 24 h, cell viability was 60% of controls, whereas protein synthesis was 20%.

Numerous studies demonstrated that RIPs induce apoptosis as main cell death pathway [41,42,43]. Kirkiin was able to trigger apoptosis showing cellular and nuclear alterations compatible with an apoptotic pattern, elevated Annexin V positivity, altered mitochondrial transmembrane potential, and strong and fast caspase 3/7 activation. Interestingly, the pan-caspase inhibitor Z-VAD caused a high rescue of cells from death after kirkiin exposure, demonstrating that the apoptotic pathway is the dominant death mechanism. However, the lack of total protection at incubation periods longer than 16 h indicates that the toxin activates other cell death mechanisms, as already described for other RIPs [25,42,44,45].

Caspase activation is an early event with respect to inhibition of protein synthesis. In fact, while caspases are significantly activated starting from 4 h after intoxication, the inhibition of protein synthesis becomes significant only starting from 16 h. These results suggest that caspase activation is independent of inhibition of protein synthesis. This phenomenon has already been described for other RIPs [25,43,46].

## 4. Conclusions

In this paper, we demonstrated that the new type 2 RIP kirkiin is a galactose-binding lectin able to efficiently inhibit protein synthesis and to agglutinate erythrocytes. In addition, kirkiin showed biochemical, enzymatic, and cytotoxic characteristics typical of type 2 RIPs, possessing N-glycosylase activity on mammalian and yeast ribosomes, but little or no activity on other nucleotide substrates. This toxin is able to completely inhibit cell protein synthesis and to induce cell death by apoptosis at very low doses. The high cytotoxicity of kirkiin, similar to that of other toxins derived from plants belonging to *Adenia* genus, represents an important opportunity for the present and future development of new drugs. Indeed, kirkiin in native form could find application for loco-regional treatments, whereas kirkiin A chain could be used as a component of immunotoxins, for systemic treatments, mainly against hematological tumors [1,47]. Moreover, the assessment of the ability of these toxins to induce apoptosis and to be transported in a retrograde manner in the central nervous system may have very interesting applications in neuroanatomy, neurophysiology, and in the study of degenerative diseases affecting muscle tissue and the nervous system.

## 5. Materials and Methods

### 5.1. Materials

*A. kirkii* caudex was purchased from Mbuyu–Sukkulenten, Bielefeld, Germany. Stenodactylin [17], volkensin [48], ricin [49], and type 1 RIPs [50] were obtained as previously described.

Human neuroblastoma-derived NB100 cell line was from long term culture in our department [25] and was maintained at the logarithmic phase of growth in Roswell Park Memorial Institute medium 1640 (RPMI-1640), supplemented with 10% (*v*/*v*) heat-inactivated fetal bovine serum, 2 mM L-glutamine, 100 U/mL penicillin G, and 100 μg/mL streptomycin (hereafter referred as complete medium) at 37 °C in a humidified atmosphere containing 5% CO_2_ in a HeraCell Haraeus incubator (Hanau, Germany). Cells were routinely checked for the absence of mycoplasma infection. Trypan Blue and trypsin/EDTA were obtained from BioWhittaker (Vervies, Belgium). L-[4,5-^3^H] leucine was purchased by GE Healthcare (Buckingamshire, UK). Flasks and plates were from Falcon (Franklin Lakes, NJ, USA). The pan-caspase inhibitor carbobenzoxy-valyl-alanyl-aspartyl-[O-methyl]-fluoromethylketone (Z-VAD-fmk, hereinafter indicated as Z-VAD) was purchased from Vinci-Biochem (Florence, Italy).

Rabbit sera against ricin, volkensin, type 1 RIPs [51], and stenodactylin [17] were prepared as previously described. The alkaline phosphatase-conjugated antirabbit IgG used for ELISA was purchased from Sigma-Aldrich (St. Louis, MO, USA); the phosphatase substrate (4-nitrophenyl phosphate disodium salt hexahydrate) was purchased from Merck (Darmstadt, Germany). 

Caspase activity was evaluated using the luminescent kit Caspase-Glo™3/7 Assay (Promega Corporation, Fitchburg, WI, USA). Morphological membrane changes were detected using Annexin V-EGFP/PI detection kit (Biovision, Mt. View, CA, USA). Viability was measured using the colorimetric CellTiter 96^®^ Aqueous One Solution Cell Proliferation Assay (Promega), which contains the tetrazolium compound [3-(4,5-dimethylthiazol-2-yl)-5-(3-carboxymethoxyphenyl)-2-(4-sulfophenyl)-2H-tetrazolium, MTS] and an electron coupling reagent (1-methoxy phenazine methosulfate, PMS). The mitochondrial potential changes were detected using the Mitochondria Staining Kit (Sigma-Aldrich).

The liquid scintillation cocktail was the Ready-Gel (Beckman Instrument, Fullerton, CA, USA).

Pre-casted gels, molecular weight standards, and buffer strips used for electrophoretic analysis were obtained from GE Healthcare. DAPI-Antifade was from Resnova SRL, Genzano di Roma, Italy. Yeast RNA was purchased from Roche Diagnostics S.L. (Barcelona, Spain). Single-stranded salmon sperm DNA was purchased from Sigma-Aldrich. The water used was prepared with a Milli-Q apparatus (Millipore, Milford, MA, USA). Other reagents used were from Merck (Darmstadt, Germany), Carlo Erba (Milano, Italy), and Sigma. All reagents were of analytical grade, and when possible RNase-free.

### 5.2. Methods

#### 5.2.1. Adenia Kirkii Lectin Purification

*A. kirkii* caudex (446 g) was decorticated and homogenized with an Ultra-Turrax (IKA, Staufen, Germany) with 5 mL/g of phosphate-buffered saline (PBS, 0.14 M NaCl containing 5 mM sodium phosphate buffer, pH 7.4). After overnight stirring at 4 °C, the extract was strained through cheesecloth and centrifuged at 18,000× *g* at 4 °C for 30 min. The supernatant (500 mL, corresponding to 1730 mg of proteins) was subjected to affinity chromatography on Sepharose CL-6B matrix (GE Healthcare), pre-treated with 0.2 M HCl for 150 min at 50 °C (acid-treated Sepharose CL-6B), and equilibrated with PBS. The sample was loaded onto the acid-treated Sepharose CL-6B column (7cm h × 5cm Ø) and, after wash with PBS to eliminate the unbound material, the retained protein was eluted stepwise with 0.2 M galactose in PBS (as already described in [17,31]). The determination of the protein content of crude extract and not retained material by acid-treated Sepharose CL-6B was performed by spectrophotometric analysis at 230, 260, and 320 nm, using the Kalb and Bernlohr method [52].

Lectins from acid-treated Sepharose CL-6B were analyzed by 8–25% sodium dodecyl sulfate-polyacrylamide gel electrophoresis (SDS-PAGE) using the PhastSystem (GE-Healthcare) both under reducing and non-reducing conditions.

The volume eluted from acid-treated Sepharose CL-6B (38 mL) was concentrated to 2 mL on YM10 membrane (Merck Millipore, Burlington, MA, USA) under nitrogen pressure and loaded into a Sephacryl S-100 column (94 cm h × 1.5 cm Ø) (GE-Healthcare) in PBS. Peak fractions of the S-100 protein peaks were analyzed on 8–25% PhastGel gradient, following the supplier’s protocol. The protein fractions corresponding to the purified lectins were collected and analyzed on a 4–15% PhastGel gradient.

For electrophoretic analysis, proteins were incubated in sample buffer (40 mM Tris/HCl pH 6.8, 2% SDS, 0.005% bromophenol blue) containing 0.5% (*v*/*v*) 2-mercaptoethanol (reducing conditions), or 1 mg/mL iodoacetamide (non-reducing conditions) for 20 min at 37 °C. The gel was stained with 0.1% (*w*/*v*) Coomassie Brilliant Blue G250 in 50% methanol and 10% acetic acid, following the protocol recommended by the manufacturer (GE Healthcare). Densitometric analysis of gels was carried out using ImageJ software, version 1.53a (National Institutes of Health, Bethesda, MD, USA). 

#### 5.2.2. Cell Free Protein Synthesis Inhibition

The effect of lectins on protein synthesis was determined through a cell-free system, based on a rabbit reticulocyte lysate. Experiments were carried out both under non-reducing and reducing conditions with the addition of 1% 2-mercaptoethanol for 30 min at 37 °C. Samples were diluted and added to the reaction mixture, as previously described [53]. The radioactivity of L-[^3^H]leucine incorporated into new synthesized proteins was measured by β-counter (Beckman Instruments). The experiments were conducted in duplicate, and IC_50_ values were calculated by linear regression. Specific activity is expressed as units (U) per mg of protein, where one U is the amount of proteins (in μg) that inhibits 50% protein synthesis in 1 mL of reaction mixture. Total activity was calculated as the specific activity per whole basic-fraction proteins (mg) normalized to the total proteins of the crude extract.

#### 5.2.3. Hemagglutinating Activity

Hemagglutinating activity was determined in 96 wells microtiter plates. Each well contained 50 μL of a 2% suspension of human erythrocytes (group 0, Rh+) and 2-fold serial dilutions of the lectins, in a final volume of 100 μL. The plates were gently shaken and after about 1 h at 25 °C, the presence/absence of agglutination was visually examined.

#### 5.2.4. rRNA Glycosylase Activity

N-glycosylase activity of kirkiin was conducted as previously described [54]. Briefly, rabbit reticulocytes lysate (40 μL) and S-30 lysate from yeast (25 μL) were incubated with 3 μg of kirkiin at 37 °C for 1 h. After treatment, 2 μL of 0.5 M EDTA pH 8 and 500 μL of 50 mM Tris-HCl (pH 7.8) and 0.5% SDS (*w*/*v*) were added, and the samples were vigorously vortexed for 30 s. RNA was extracted by phenolization, treated with 2 M aniline acetate (pH 4.5) on ice for 10 min in the dark, and precipitated with ethanol. The pellet was resuspended in 20 μL of sterile water and the concentration was determined by spectrophotometer at 260 nm. Ribosomal RNA was analyzed using 5% (*w*/*v*) polyacrylamide in denaturating conditions with 7 M urea. RNA samples were incubated in loading buffer containing 150 mg/mL sucrose, 7 M urea, 0.4 μg/mL bromophenol blue, and 1XTBE buffer (45 mM Tris, 45 mM boric acid, 1 mM EDTA pH 8). After boiling the samples for 30 s, the run was performed at 15 mA for 1 h 50 min, approximately, using TBE buffer. The gel was stained with ethidium bromide (20 mg/mL) in TBE buffer for 20–30 min and RNA bands were analyzed by UV-transilluminator (254–312 nm) including in the imaging instrument GelDoc (Biorad).

#### 5.2.5. Adenine Polynucleotide Glycosylase Activity on Salmon Sperm DNA and on Tobacco Mosaic Virus (TMV) RNA

Adenine polynucleotide glycosylase activity was determined by measuring the adenine release from salmon sperm DNA (ssDNA) according to the method reported in [55] with a few modifications. Briefly, 10 μg of ssDNA were incubated with 5 μg of kirkiin, both in reduced and non-reduced conditions, in 300 μL of a reaction mixture containing 1 M KCl and 0.5 M sodium acetate (pH 4.5) at 30 °C for 1 h. After incubation, the DNA was precipitated with ethanol at −80 °C overnight and centrifugated at 13,000 rpm for 15 min at 4 °C. Adenine released from RIP-treated DNA was determined in the supernatants by spectrophotometer at 260 nm.

On TMV, the adenine polynucleotide glycosylase activity of kirkiin was assayed as described in [54]. Briefly, 25 μL samples containing 15 μg of TMV RNA were incubated with 3 μg of kirkiin. After treatment, the RNA was analyzed by extraction, phenolization, treatment with 2 M aniline acetate (pH 4.5), and ethanol precipitation. The RNA was subjected to electrophoresis on 5% (*w*/*v*) polyacrylamide-7 M urea gel at 15 mA for 75 min and stained with ethidium bromide.

#### 5.2.6. Endonuclease Activity on Supercoiled Plasmid DNA

The endonuclease activity of the RIP was assayed on the *E. coli* plasmid pCR 2.1 (Invitrogen). 200 ng of the plasmid were incubated with 3 μg of kirkiin at 37 °C for 1 h in a final volume of 10 μL of 10 mM Tris-HCl (pH 7.8), 50 mM NaCl, and 50 mM KCl in presence/absence of 5 mM MgCl_2_. The samples were analyzed on 0.8% agarose gel electrophoresis in TAE buffer (0.04 M Tris, 0.04 M acetate, 1 mM EDTA, pH 8.0) and visualized by gel red staining.

#### 5.2.7. Enzyme-Linked Immunosorbent Assay (ELISA)

ELISA assay was performed as described previously [17], using 2 μg per well of kirkiin in 100 μL of 50 mM carbonate buffer pH 9.0 containing 15 mM sodium carbonate and 35 mM sodium bicarbonate. Reciprocal serum dilutions (from 1:100 to 1:12,800) were added. The dilutions were prepared in 50 mM lactose, 50 mM mannose, and 0.05% Tween 20. Rabbit antisera against type 1 and type 2 RIPs were obtained as described in [17,51]. 100 μL of anti-rabbit secondary antibody (1:7000) conjugated to alkaline phosphatase was used and incubated 1 h at 37 °C. 100 μL of 1 mg/mL enzyme substrate (4-nitrophenyl phosphate disodium) dissolved in buffer containing 1 M diethalonamine, 0.5 M MgCl_2_ × 6H_2_O, and 3 mM NaN_3_ were added. The absorption was measured at 405 nm with the Multiskan EX microtiter plate reader (ThermoLabsystem, Helsinki, Finland).

#### 5.2.8. Cell Protein Synthesis Inhibition and Viability Assay

The cytotoxicity of kirkiin was assessed by evaluating both protein synthesis inhibition and viability reduction.

Protein synthesis inhibition was evaluated through L-[^3^H]leucine incorporation in neosynthesized proteins. NB100 cells (2 × 10^4^/well) were seeded onto 24-well plates in 250 µL of complete medium in the absence (control cultures) or presence of scalar dilutions (from 1 × 10^−15^ to 1 × 10^−11^ M) of kirkiin. After 72 h, cell protein synthesis was evaluated as previously described [18]. In addition, time course experiments were conducted on cells exposed to kirkiin (1 × 10^−11^ M), in a range between 4 and 48 h. The IC_50_ and IT_50_ (kirkiin concentration and time required to inhibit cell protein synthesis by 50%) were calculated using linear regression analysis.

Cell viability was evaluated through the colorimetric cell cytotoxicity assay (CellTiter 96^®^ Aqueous One Solution Cell Proliferation), based on the cellular conversion of a tetrazolium salt into a colored formazan. NB100 cells (2 × 10^3^/100 μL complete medium) were seeded in 96-well microtiter plates. After 24 h, cells were incubated with scalar dilutions of kirkiin (from 1 × 10^−15^ to 1 × 10^−11^ M) and left for 72 h. In addition, time course experiments were conducted on cells exposed to kirkiin (1 × 10^−11^ M), in a range between 4 and 48 h. After the indicated times, the medium was removed and CellTiter 96 Aqueous One Solution Reagent (1: 6 in complete medium). After 1 h of incubation at 37 °C, the absorbance at 492 nm was measured. The EC_50_ and ET_50_ (kirkiin effective concentration and time required to reduce cell viability by 50%) were calculated using linear regression analysis. The results are the means of at least three experiments performed in triplicate.

#### 5.2.9. Evaluation of Apoptosis

The morphological analysis of treated cells (2 × 10^3^/100 μL complete medium) was conducted through phase contrast microscopy, directly in 24-well plate, using an inverted microscope Nikon Eclipse TS100 (Nikon, Melville, NY, USA). For the nuclear analysis, NB100 cells (2 × 10^4^/500 μL complete medium) were seeded directly on a coverslip in 24-well plates 48 h prior to the experiment. After treatment with kirkiin for 48 h, cells were fixed with methanol/acetic acid 1:3 for 20 min. The analysis was conducted under Nikon Eclipse E600W fluorescence microscope with pretreatment of cells with 7 μL DAPI/antifade (4′,6-diamidino-2-phenylindole).

The mitochondrial membrane potential (Δѱm) was measured using the cationic, lipophilic dye JC-1 (5,5′,6,6′-tetrachloro-1,1′,3,3′-tetraethylbenzimidazolocarbocyanineiodide) contained in Mitochondria Staining Kit (Sigma). JC-1 selectively enters the mitochondria and reversibly change color from green to red as the membrane potential increases. Cells (2 × 10^4^/500 μL complete medium) were seeded directly on a coverslip in 24-well plates 48 h prior the experiments. After treatment with kirkiin for 48 h, cells were stained with 500 μL of JC-1 dye (1:100 in complete medium) and incubated at room temperature for 10 min in the dark. The cells were then washed three times with staining buffer purchased from the kit. The coverslips were inverted on glass slide and the cells were observed under Nikon Eclipse E600W fluorescence microscope.

Apoptotic cell death was assessed using a flow cytometry Annexin V-EGFP/PI detection kit and by a luminescent reagent detecting caspase activity. Before flow cytometry, cells (2 × 10^6^/3 mL complete medium) were seeded in 25 cm^2^ flasks, and after incubation with kirkiin for 48 h, the cells were pelleted at 400× *g* for 5 min, washed twice in cold PBS, pelleted, and resuspended in 294 µL of binding buffer provided by the kit. Annexin V-EGFP (3 µL) and PI (3 µL) were added. After 10 min incubation in the dark at room temperature, cells were analyzed by flow cytometry FACSAria (BD) using the FACSDiva software.

The caspase-3/7 activity was assessed by the luminescent Caspase-Glo™3/7 Assay as described in [25]. Briefly, cells (2 × 10^3^/100 µL complete medium) were seeded in 96-well microtiter plates. After incubation with kirkiin for the indicated amounts of time, 50 µL/well of caspase kit reagent (1:2 in complete medium) was added. The plates were shaken at 420 rpm for 1 min and then incubated for 20 min at room temperature in the dark. The luminescence was acquired (integration time 10 s) by a Fluoroskan Ascent FL (Thermo Labsystems) and the values were normalized for cell viability.

#### 5.2.10. Statistical Analyses

Statistical analyses were conducted using XLSTAT-Pro software, version 6.1.9, 2003 (Addinsoft, Inc., Brooklyn, NY, USA). The results are presented as the means ± S.D. of three different experiments. The data were analyzed using ANOVA/Bonferroni test or Student’s *t*-distribution. The Dunnett’s test was used in addiction to ANOVA, when necessary.

## Figures and Tables

**Table 1:** Biological activity of *Adenia* toxic lectins purified by chromatography on acid-treated Sepharose CL-6B.

*Adenia* Species	Cell Free IC_50_ (μg/mL)		Agglutinating Activity ^1^ (μg/mL)	Ref.
	Non-Red.	Red.		
*A. kirkii*	9.2	4.7	4.0	
*A. stenodactyla*	5.6	0.5	49.9	[17]
*A. lanceolate*	5.2	1.1	230.9	[17]
*A. volkensii*	7.5	0.7	15.6	[15,17]

^1^ Minimum concentration causing hemagglutination.

**Table 2:** *Adenia kirkii* lectins purification summary.

Purification Step	Protein (mg/mL)	Total Protein (mg)	Total Protein (%)	IC_50_ (µg/mL) ^1^		Agglutinating Activity ^2^ (μg/mL)	Specific Activity (U/mg) ^3^	Total Activity (U)	Yeld (%)
				Non-Red.	Red.				
Crude extract	3.46	1730.0	100.0	55.0	21.2	45.1	18.2	31,454.5	100.0
Sepharose CL-6B Eluate	2.84	107.9	6.2	9.2	4.7	4.0	108.7	4130.4	13.1
Sephacryl S-100 peak 1	1.43	24.2	1.4	7.4	1.0	175.0	135.1	3270.2	10.4
Sephacryl S-100 peak 2	1.49	62.4	3.6	>50	>50	2.9	-	-	-

^1^ Concentration of protein that inhibits the 50% of protein synthesis in a cell free system, measured by linear regression. ^2^ Minimum concentration causing hemagglutination. ^3^ Units of IC_50_ (non-reducing conditions) in 1 mg of protein.

## Data Availability

Data are available upon request. Please, contact the contributing authors.
